# Supplementary material for: Effects of Butyrate Supplementation on Inflammation and Kidney Parameters in Type 1 Diabetes: A Randomized, Double-Blind, Placebo-Controlled Trial
Source: J Clin Med. 2022 Jun 21;11(13):3573. doi: 10.3390/jcm11133573 (PMC9267418; doi:10.3390/jcm11133573)
Supplement: Supplementary file 1 [file jcm-11-03573-s001.zip › jcm-1703562-supplementary.pdf]

Table S1. Metabolite panel.

	Name	Class
a(R)-OHB	1. 2-Hydroxybutyric acid	Small organic acids
b-OHB	2. 3-Hydroxybutyric acid	
GBB	3. 4-Trimethylammoniobutanoic acid	
AzelA	4. Azelaic acid	
AADA	5. Amino adipic acid	
TMAO	6. Trimethylamine N-oxide	Amine oxide, amides, sulfates
N-MNA	7. N-methylnicotinamide	
IndS	8. Indoxyl sulfate	
ADMA	9. Asymmetric dimethylarginine	Amino acids
Gly	10. Glycine	
Taurine	11. Taurine	
Gln	12. L-Glutamine	
Glu	13. L-Glutamic acid	
Cit	14. Citrulline	
HCit	15. Homocitrulline	

Ala	16. L-Alanine	
Tyr	17. L-Tyrosine	
Leu	18. L-Leucine	
Kynu	19. L-Kynurenine	
Ile	20. L-Isoleucine	
Phe	21. L-Phenylalanine	
Trp	22. L-Tryptophan	
Crea	23. Creatinine	
TCA	24. Taurocholic acid	Bile acids
TCDCA	25. Taurochenodesoxycholic acid	
GCA	26. Glycocholic acid	
GUDCA	27. Glycoursodeoxycholic acid	
GCDCA	28. Glycodeoxycholic acid	
CDCA	29. Chenodeoxycholic acid	
UDCA	30. Ursodeoxycholic acid	
GDCA	31. Glycochenodeoxycholic acid	
TLCA	32. Taurolithocholic acid	
CA	33. Cholic acid	

LCA	34. Lithocholic acid	
TDCA	35. Taurdeoxycholic acid	
GLCA	36. Glycolithocholic acid	
DCA	37. Deoxycholic acid	
TUDCA	38. Tauroursodeoxycholic acid	

Table S2. Change in serum metabolites after 12 weeks intervention compared by ANCOVA.

	Before	After	Change	ANCOVA p
2-Hydroxybutyric acid (ng/mL)				
• Sodium Butyrate	22,377 ± 3332	21,878 ± 3218	-499 ± 4359	0.37
• Placebo	22,062 ± 3177	22,808 ± 4476	602 ± 4881	
3-Hydroxybutyric acid (ng/mL)				
• Sodium Butyrate	13,990 ± 1624	14,680 ± 2488	689 ± 3253	0.23
• Placebo	14,688 ± 2620	15,459 ± 1746	807 ± 2773	
4-Trimethylammoniobutanoic acid (ng/mL)				
• Sodium Butyrate	386 [323:461]	427 [327:516]	18 [-40:99]	0.09
• Placebo	442 [372:588]	405 [290:523]	-6 [-128:51]	
Trimethylamine N-oxide (ng/mL)				
• Sodium Butyrate	248 [209:497]	257 [217:333]	13 [-112:144]	0.06
• Placebo	276 [184:409]	222 [172:292]	-12 [-151:32]	
Asymmetric dimethylarginine (ng/mL)				
• Sodium Butyrate	4879 ± 1534	4809 ± 1616	-70 ± 2308	0.93
• Placebo	4874 ± 1205	4776 ± 1372	-236 ± 1535	

Glycine (ng/mL)				
• Sodium Butyrate	6521 [4847:8973]	8440 [6820:10084]	539 [-1938:3803]	0.49
• Placebo	7705 [5637:10079]	6977 [5229:9143]	-936 [-5080:4144]	
Taurine (ng/mL)				
• Sodium Butyrate	4861 [3478:6492]	5621 [3458:6544]	277 [-1885:2653]	0.67
• Placebo	4919 [3981:6730]	5010 [3844:7345]	-375 [-2792:2910]	
L-Glutamine (ng/mL)				
• Sodium Butyrate	426,308 [281,386:547,389]	487,750 [342,208:586,095]	55,681 [-159,487 :257,062]	0.98
• Placebo	394,754 [307,282:621,025]	490,443 [309,685:759,158]	23,986 [-117,143:318,692]	
L-Glutamic acid (ng/mL)				
• Sodium Butyrate	24,685 ± 12,736	27,776 ± 11,393	3091 ± 16,641	0.81
• Placebo	27,828 ± 12,651	27,347 ± 14,002	196 ± 16,006	
Citrulline (ng/mL)				
• Sodium Butyrate	3247 [2169:5325]	4546 [2487: 6954]	853 [-1032:2929]	0.59
• Placebo	3841 [3107:5701]	4705 [2891:5984]	-104 [-1833:2320]	
Homocitrulline (ng/mL)				
• Sodium Butyrate	250 [141:365]	265 [178:388]	43 [-225:163]	0.30

• Placebo	301 [166:453]	326 [169:477]	-4 [-138:237]	
L-Alanine (ng/mL)				
• Sodium Butyrate	26,725 [16,867:35,115]	33,564 [22,102:43,648]	5181 [-3959:23080]	0.76
• Placebo	29,161 [23,155:38,957]	27,874 [22,176:48,967]	-372 [-11,099:15,677]	
L-Tyrosine (ng/mL)				
• Sodium Butyrate	4401 [2731:6630]	5740 [3149:8306]	1031[-1410: 3417]	0.37
• Placebo	4500 [3456:6902]	5161 [3222:8695]	100 [-2527:2842]	
Azelaic acid (ng/mL)				
• Sodium Butyrate	275 [251:295]	289 [270:308]	16 [-12:40]	0.49
• Placebo	258 [246:282]	276 [257:297]	22 [-14:50]	
L-Leucine (ng/mL)				
• Sodium Butyrate	9931 [6840:15609]	12,560 [8755:15,851]	1669 [-4304:7083]	0.58
• Placebo	10,416 [9042:12,052]	12,513 [7716:19387]	2130 [-5378:9095]	
L-Kynurenine (ng/mL)				
• Sodium Butyrate	891 [621:1097]	909 [739:1308]	121 [-79:375]	0.91
• Placebo	936 [740:1186]	937 [696:1281]	44 [-139:353]	
L-Isoleucine (ng/mL)				
• Sodium Butyrate	8203 [5702:10702]	12,129 [7079:14,194]	2290 [-3127:6525]	0.77

• Placebo	8452 [7882:12,161]	10,563 [7265:16,200]	-485 [-4222:6930]	
L-Phenylalanine (ng/mL)				
• Sodium Butyrate	8617 [6221:12,174]	10,527 [7750:13,269]	802 [-2120:5855]	0.82
• Placebo	9394 [7809:13,453]	9919 [7547:14,331]	769 [-4270:6418]	
L-Tryptophan (ng/mL)				
• Sodium Butyrate	6757 [4355:9560]	7718 [5384:11,152]	1292 [-2362:4200]	0.88
• Placebo	7465 [5405:9705]	7484 [5577:11,513]	690 [-2952:3581]	
Taurocholic acid (ng/mL)				
• Sodium Butyrate	261 [179:414]	294 [231:464]	55 [-13:230]	0.64
• Placebo	334 [249:521]	384 [172:801]	6 [-115:217]	
Taurochenodesoxycholic acid *				
• Sodium Butyrate	5.9 [5.2:8.3]	8.4 [5.7:12]	1.8 [-1.8:5.5]	0.48
• Placebo	8.3 [6.7:12]	8.0 [3.8:13]	-0.04 [-5.4:3.7]	
Glycocholic acid (ng/mL)				
• Sodium Butyrate	357 [230:552]	386 [281:563]	14 [-94:156]	0.55
• Placebo	375 [241:529]	376 [196:649]	-6.8 [-267:225]	
Glycoursodeoxycholic acid (ng/mL)				
• Sodium Butyrate	22 [21:26]	26 [22:29]	1.7 [-2.5:6.5]	0.14

• Placebo	24 [22:29]	25 [21:30]	-0.60 [-4.8:2.2]	
Glycodeoxycholic acid (ng/mL)				
• Sodium Butyrate	395 [369:412]	397 [347:423]	5.1 [-49:37]	0.42
• Placebo	401 [372:465]	400 [369:454]	-4.5 [-118:44]	
Chenodeoxycholic acid *				
• Sodium Butyrate	196 [184:220]	211 [193:235]	17 [-15:50]	0.42
• Placebo	207 [181:237]	205 [178:225]	9.5 [-31:38]	
Ursodeoxycholic acid (ng/mL)				
• Sodium Butyrate	153 [146:158]	155 [149:169]	2.8 [-2.9:20]	0.71
• Placebo	156 [148:170]	163 [149:170]	7.9 [-30:21]	
Glycochenodeoxycholic acid (ng/mL)				
• Sodium Butyrate	48 [35:70]	48 [34:76]	-0.41 [-13:14]	0.27
• Placebo	50 [42:84]	45 [32:61]	-19 [-44:4.3]	
Taurolithocholic acid *				
• Sodium Butyrate	22,762 [16,804:28,307]	24,118 [17,165:27,228]	-744 [-11,656:10,422]	0.34
• Placebo	25,126 [19,653:33,056]	20,995 [16,199:25,347]	-4171 [-9376:-418]	
Cholic acid (ng/mL)				
• Sodium Butyrate	195 [138 :512]	200 [143:318]	-8 [-299:98]	0.22

• Placebo	202 [148:397]	174 [31:321]	-12 [-176:30]	
Lithocholic acid (ng/mL)				
• Sodium Butyrate	106 ± 28	96 ± 24	-10 ± 30	0.71
• Placebo	97 ± 22	98 ± 36	0.42 ± 43	

Values are mean ± standard deviation or median [Q1:Q3]. Parameters with a non-normal distribution were log transformed before analysis. P value for the group-wise comparison of participants treated with sodium butyrate or placebo was calculated using baseline-corrected linear regression.

*Calculated concentrations were below limit of quantification and values are represented by the peak area of the compound in the chromatogram obtained in the samples via UHPLC-MS analysis.

Table S3. Gastrointestinal symptoms before and after 12 weeks intervention.

	Baseline			Improvement of existing symptom		Worsening or onset of symptom	
	ALL	Sodium butyrate	Placebo	Sodium butyrate	Placebo	Sodium butyrate	Placebo
Diarrhea	16/51 (31%)	7 /28	9/23	2/7 (29%)	6/9 (67%)	1/28 (4%)	2/23 (9%)
Constipation	14/51 (27%)	6/28	8/23	5/6 (83%)	4/8 (50%)	3/28 (11%)	2/23 (9%)
Abdominal pain	13/51 (25%)	8/28	5/23	5/8 (63%)	2/5 (40%)	2/28 (7%)	5/23 (22%)
Heartburn	10/51 (20%)	6/28	4/23	1/6 (17%)	1/4 (25%)	3/28 (11%)	6/23 (26%)
Nausea	10/51 (20%)	5 /28	5/23	4/5 (80%)	5/5 (100%)	2/28 (7%)	2/23 (9%)
Poor appetite	9/47 (19%)	5 /25	4/22	2/5 (40%)	4/4 (100%)	2/25 (8%)	2/22 (9%)
Vomiting	1/51 (2%)	1/28	0/23	0/1 (0%)	0/0 (-)	0/1 (0%)	0 (-)

Proportions of participants who reported gastrointestinal symptoms within two weeks before baseline and how the symptoms were reported within two weeks before EOS. The numbers do not discriminate between the frequency and severity of the symptom.
